# Risk and Protective Factors for Breast Cancer in Midwest of Brazil

**DOI:** 10.1155/2012/356851

**Published:** 2012-05-15

**Authors:** Lívia Emi Inumaru, Maíra Irineu Gomes Duarte Quintanilha, Érika Aparecida da Silveira, Maria Margareth Veloso Naves

**Affiliations:** ^1^Nutrition Faculty, Federal University of Goiás, Rua 227, Quadra 68, s/n°, Setor Leste Universitário, 74605-080 Goiânia, GO, Brazil; ^2^Department of Agricultural, Food and Nutritional Science, University of Alberta, 4-10 Agriculture/Forestry Centre, Edmonton, AL, Canada T6G 2P5

## Abstract

Patterns of physical activity, body composition, and breastfeeding are closely related to health and are influenced by environmental, economic, and social factors. With the increase of sedentary lifestyle and overweight, many chronic diseases have also increased, including cancer. Breast cancer is the most common cancer in women worldwide, and the knowledge of its risk and protective factors is important to the adoption of primary prevention strategies. We aimed to investigate some risk and protective factors for breast cancer among women from Midwest Brazil. It is a case-control study of outpatient basis, carried out with 93 breast cancer cases and 186 controls. Socioeconomic, gynecological, anthropometric, and lifestyle variables were collected, and odds ratios (ORs) values were estimated (significance level, 5%; confidence interval (CI), 95%). Per capita income equal to or lower than 1/2 Brazilian minimum wage (OR = 1.88; CI = 1.06–3.29), residence in rural area (OR = 4.93; CI = 1.65–14.73), and presence of family history of breast cancer (OR = 5.38; CI = 1.46–19.93) are risk factors for breast cancer. In turn, physical activity (past 6 months) (OR = 0.23; CI = 0.10–0.55) and leisure physical activity at 20 years old (OR = 0.13; CI = 0.03–0.54) are protective factors for the disease in women who live in Midwest of Brazil.

## 1. Introduction

Around the world, populations have changed rural areas for cities, becoming more sedentary and consuming great amounts of industrialized foods and beverages, which have contributed for the increase of obesity and chronic diseases, such as cancer. These changes began in Europe, North America, and other countries that became industrialized in XIX century. In recent decades, there was acceleration in urbanization process around the world, especially in low-middle-income countries, like Brazil. Patterns of physical activity, body composition, and breastfeeding are determined by environmental (urbanization, urban planning, which includes transport systems), economic (social status), and social (education level) factors, influencing, thus, the occurrence of several types of cancer [1, 2].

Breast cancer is the most common cancer in women worldwide and has been associated with higher socioeconomic status and urbanized populations [2, 3]. Family history of breast cancer and gynecological variables, such as early menarche, late menopause, not bearing children, and late first pregnancy are well established risk factors for breast cancer [2, 4]. Besides these variables, according to the World Cancer Research Fund (WCRF) and the American Institute for Cancer Research (AICR) [2], drinking alcoholic beverages is a risk factor, whereas breastfeeding is a protective factor for breast cancer. However, regarding the body fatness, adult attained height, abdominal fatness, adult weight gain, and physical activity, the evidences are not sufficiently clear [2]. Body fatness is a convincing risk factor only for postmenopausal breast cancer, and the protective influence of physical activity is not convincing yet [2].

Most studies on the determinant factors for breast cancer, especially related to physical activity, have been conducted in high-income countries [5–8]. Thus, some risk factors pointed by the WCRF and the AICR [2] may be different from that found in low-middle-income countries, such as Brazil, which has a different epidemiological profile. In most of Latin American countries, the transition experience is unlike that of the developed countries and is distinguished by the coexistence of infectious diseases and chronic diseases as important causes of death. This epidemiological profile is called “transition polarized model” [9].

Furthermore, in Brazil and other Latin American countries, there are few recent analytical studies on the risk and protective factors for breast cancer. According to a 2009 report by Brazilian National Cancer Institute [4], in Goiânia, capital of Goiás state (Midwest of Brazil), the estimated risk for breast cancer was 51.87 new cases per 100 000 women, which was close to the national mean estimate (49.27 new cases per 100 000 women). Knowledge of risk and protective factors among populations is important to identify groups that are more susceptible and to develop strategies for primary prevention [10]. Thus, the objective of this study was to investigate some risk and protective factors for breast cancer among women from Midwest of Brazil and with recent diagnosis of breast cancer.

## 2. Material and Methods

### 2.1. Material

This outpatient-based case-control study was conducted in 2 reference public hospitals of Goiânia, capital of Goiás state, situated in Midwest of Brazil. Cases were interviewed at the Conjunctive Tissue Sector and Gynecology and Breast Clinic of Araújo Jorge Hospital (HAJ), a public institution that specializes in the prevention, diagnosis, and treatment of cancer, that provides health service to people from throughout Midwest of Brazil. Cases were also interviewed at the Breast Service of the General Hospital of the Federal University of Goiás (Hospital das Clínicas da Universidade Federal de Goiás, HC/UFG). Controls were selected among users of the Gynecology Clinic at HC/UFG. Both hospitals (HAJ and HC/UFG) attend to Unique Health System (SUS) users, which have a similar socioeconomic profile.

To calculate the sample size, the following criteria were considered: significance level of 5%, study power (1-*β*) of 80%, 2 controls for each case, an odds ratio (OR) of 2.5 [12], and a prevalence of risk for consumption of alcohol among controls of 14.4% [13]. According to these parameters, the total sample must include 186 controls and 93 cases of women with breast cancer. The inclusion and exclusion criteria (cases and controls) are shown in Table 1.

This study was approved by the ethics committees of UFG (protocol no. 026/08) and Araújo Jorge Hospital (protocol no. 019/08). Written informed consent was obtained from all participants.

### 2.2. Data Collection

Data were collected between May 2008 and June 2010, through a questionnaire that had been previously tested in a pilot study. This questionnaire included socioeconomic variables (per capita income—in American dollar—education level, and area of residence), family history of cancer and breast cancer, gynecological variables (number of children, age at first pregnancy, age of menarche, and age of menopause), breastfeeding information, anthropometric measures, alcoholic drink consumption, physical activity, and smoking habit. Questions about alcoholic drink consumption, physical activity, and smoking habit were asked regarding the 6 months prior to the interview date. Data on body weight and physical activity were also investigated considering the young age (20 years old) [14].

Interviewees provided data on occurrence and duration of breastfeeding (in months) for each of their children, which were added up to provide lifetime duration of breastfeeding. Anthropometric variables included height, current and previous (at 20 years-old) body weight, waist circumference, current and previous body mass index (BMI), and adult weight gain. The first 3 variables were obtained according to the protocol reported by Lohman et al. [15]. Before data collection, these measures were standardized among interviewers, according to Habicht [16]. The cut-off points used to classify nutritional status and waist circumference were established based on the World Health Organization [11]. Weight gain in adulthood was estimated by the difference between weight at the time of research and weight at 18–20 years old [14].

The daily intake of ethanol was calculated as follows: the amount of each alcoholic beverage consumed per week was multiplied by its alcohol content and divided by 7 [17]. We considered only beer, because it was the only alcoholic beverage reported by consumers. The following parameters were adopted: a can of beer equivalent to 350 mL; a bottle, 600 mL; a glass, 250 mL. We considered the alcohol content of beer to be 5 per cent (5 g/100 mL) [18] and consumption of more than 15 g of ethanol/day as excessive consumption [2].

The subjects were questioned about physical activity (past 6 months) and were classified into 4 categories: active (moderated activities ≥30 minutes/session and ≥5 days/week, or vigorous activities ≥20 minutes/session and ≥3 days/week, or any activity adding up ≥150 minutes/week and ≥5 days/week); irregularly active A (any physical activity ≥150 minutes/week or ≥5 days/week); irregularly active B (any physical activity, but did not reach any criteria recommendation related to frequency and duration); sedentary (did not practice any physical activity at least 10 continuous minutes during the week). This classification was defined according to the eighth version of the International Physical Activity Questionnaire (IPAQ), validated to Brazilian adult women [19]. We also evaluated previous physical activity (at 20 years old), considering the practice or not of occupational, housework, and leisure physical activities.

We considered smokers as those who smoked regularly for at least 1 year [20] and ex-smokers as those who had stopped smoking at least 2 years before the interview date [17].

### 2.3. Statistical Analysis

Data were analyzed using Statistical Software for Professional 8.0 (Stata Corp., College Station, TX, USA). The normality test for quantitative variables was performed using normal curves in the Stata software. Variables were analyzed in the whole sample, without considering the menopausal status, like found in some studies [21–23], and because some controls did not know about their menopausal status, due to the surgical extraction of their womb.

In bivariate analyses, to compare the 2 groups (case and control) in relation to quantitative variables, we applied Student's *t*-test (parametric distribution) and the Mann Whitney test (nonparametric distribution). Regarding the qualitative variables, we used the chi-square test or Fisher's exact test to compare case and control groups, according to frequency distribution. For all variables related to outcome, we calculated the Wald statistic and the odds ratio (OR), with a significance level of 5% and a confidence interval (CI) of 95%. All variables with a *P* value lower than 0.20 for bivariate analyses were included in multivariate analyses. Logistic regression was performed using a hierarchical model structured in 2 levels. Socioeconomic variables, such as education level, per capita income, and area of residence were included in the first level (distal). Height, physical activity, family history of cancer and of breast cancer, bearing children, and number of children were grouped in the second level (proximal). Bearing children and number of children, although considered as demographic variables, were included in the second level because they are influenced by socioeconomic status. Variables whose *P* value was lower than 0.05 remained in the logistic regression model [24].

## 3. Results

 We invited 98 cases and 186 controls to participate in research, but 5 cases (5.1%) refused to be interviewed, adding up 279 women (93 cases and 186 controls). The average age among cases was 51.93 years (range 30–83 years) and that among controls was 51.73 years (range 28–81 years). There were no significant differences between the case and control groups in relation to socioeconomic, gynecological, and anthropometric variables. The median family income per capita was lower than the Brazilian minimum wage (R$ 510 per month, approximately US$ 300), being US$ 147 for cases (range US$ 0–1,470) and US$ 200 for controls (range US$ 0–1,029) (Table 2).

Education level, per capita income, area of residence, family history of any kind of cancer, and family history of breast cancer in the first degree were significantly associated with breast cancer in the bivariate analysis (Table 3).

Anthropometric and lifestyle variables, such as alcohol consumption, smoking habit, and physical activity, are shown in Table 3. Physical activity (past 6 months) and previous leisure physical activity (at 20 years old) were associated significantly with breast cancer in the bivariate analysis (Table 4).

We considered the following variables in the multivariate analysis: education level, per capita income, area of residence, bearing children, number of children, family history of cancer (any type), history of breast cancer, height, physical activity (past 6 months), and previous occupational and leisure physical activities. After adjusting the OR values, per capita income, area of residence, family history of breast cancer, physical activity (past 6 months), and previous leisure physical activity remained associated with breast cancer (Table 5).

Per capita income equal to or lower than 1/2 Brazilian minimum wage (OR = 1.88; 95% CI = 1.06–3.29), residence in rural area (OR = 4.93; 95% CI = 1.65–14.73), and presence of family history of breast cancer (OR = 5.38; 95% CI = 1.46–19.93) were considered risk factors for breast cancer. Active women were at lower risk of developing breast cancer, compared to sedentary women (OR = 0.23; CI = 0.10–0.55), and that ones who had practiced leisure physical activity at young age (20 years-old) were less likely to develop the disease, compared to who had ever practiced (OR = 0.13; CI = 0.03–0.54) (Table 5).

## 4. Discussion

The sample was very homogeneous concerning socioeconomic, anthropometric, and gynecological variables (Table 2). This can be explained by the fact that all interviews were conducted with users of public hospitals, which serve a quite similar population. Women in this sample are of low socioeconomic status, which makes up 29.3% of Brazilian Midwest families with a per capita income of 0.5–1.0 minimum Brazilian wage [25].

In Table 6, we have summarized the findings of the present study, comparing to WCRF and the AICR evidences [2]. Although nutritional status was not significantly associated with breast cancer (Tables 3 and 5), it is important to emphasize that the prevalence of current overweight (BMI ≥25 kg/m²) in both groups (cases and controls) was greater than 60%, and the prevalence of obesity (BMI ≥30 kg/m^2^) was approximately 28% among cases and 24% among controls. These values are higher than those found in Brazil, since the proportion of obesity in adult Brazilian women, between 2008 and 2009, was 16.9% [26]. Obesity statistic found in this sample is troubling, because excessive body fat can promote chronic diseases such as type 2 diabetes and increase the level of proinflammatory substances that may enhance carcinogenesis [2]. The prevalence of excessive alcoholic drinking intake was low, under 3%, for both cases and controls (Table 4), and may be related with the low socioeconomic status of the sample.

In the present study, we found that having a low per capita income (under 1/2 Brazilian minimum wage) and living in a rural area were risk factors for breast cancer (Table 5). One possible explanation for these findings may be the lesser access to health care and health information of poor women who live in rural areas. Nevertheless, previous studies report that breast cancer is positively associated with urbanization and high socioeconomic status [2–4]. These associations are mainly due to variation in known risk factors, such as late pregnancy and not bearing children, found in women from urban and high-income areas [3].

It should be added that approximately 2/3 of Brazilian population is exposed to pesticides, and the most vulnerable group to this exposure is rural workers [27]. According to Peres, Peres et al. [27], rural workers have low health risk perception related to the use of pesticides. The indiscriminate use of these substances by the rural population could explain the occurrence of some cancers. Moreover, in most Brazilian inner cities, including from Goiás State, a large proportion of women is from rural area, where there are no reference breast services. So that, suspected breast cancer cases move up to capital cities to receive health attendance. Furthermore, it should be emphasized that Goiânia's urbanization process was initiated in 1950 and occurred quickly, which was called “demographic explosion.” However, this urbanization was not followed by industrialization [28]. Thus, this population does not have the same characteristics as urban areas in developed countries. Besides, the Brazilian epidemiological transition model, called “transition polarized model,” is different from the high-income countries models, named “classic or occidental transition model.” In the “transition polarized model,” both chronic and infectious diseases coexist as important cause of death, and the decrease in mortality rate is slower. In the “classic or occidental transition model,” there is the prevalence of chronic diseases and the mortality rate decreases progressively. Therefore, risk factors may differ according to the epidemiological profile of each population [9, 29].

We also found that family history of breast cancer in first degree is a risk factor for breast cancer (Table 5), and this result is reinforced by other ones [12, 30]. According to the “theory of two stages” in the genetic determinism of carcinogenesis, it is necessary that mutations occur at the same locus of homologous chromosomes for the development of a malignant tumor. Thus, in nonhereditary cases (also called sporadic cases), 2 mutagenic processes are required, whereas, in hereditary cases, 1 genetic mutation already exists and only 1 more mutation in the other chromosome is necessary [31]. According to Brandt et al. [12], 15–20% of breast cancer cases are associated with family history of breast cancer, but only 15% can be explained by mutations in the *BRCA1* and *BRCA2* genes, known tumor suppressors. A woman who carries a mutation in *BRCA-1* has a risk of 60–80% for developing breast cancer [12, 31].

Regarding physical activity, we found that women who practiced at least 150 minutes/week and/or ≥5 days/week of physical activity were at lower risk of developing breast cancer, compared to sedentary women. Moreover, that ones who practiced leisure physical activity at young age (20 years old) were also at lower risk, compared to that women who did not practice leisure activity. These results show that physical activity, in general, may protect against breast cancer. This protective effect is supported by WCRF and AICR's report and by other case-control studies [2, 5–8]. This result is important because WCRF and AICR [2] concluded that the protective effect of physical activity is not convincing yet (probable in postmenopausal women, but limited in premenopausal women), and our study supports this evidence, which may increase the consistency of this association (Table 6).

Regular physical activity probably has a protective effect on breast cancer by delaying menarche, promoting irregular and anovulatory cycles, reducing serum estrogen, increasing globulins that bind to sex hormones, reducing inflammation, improving immune function, and helping to control weight and to improve sensitivity to insulin [2, 6]. Considering the importance of physical activity practice in prevention of breast cancer and other chronic diseases, and that health is a determinant key of development and a precursor of economic growth, it is necessary to perform programs aimed at promoting health lifestyle. To implement these policies, it is necessary the collaboration between the health sector and other key sectors, such as education, urban planning, transportation, and communication [32].

 The lack of an association of some variables with breast cancer can be explained partially by the recall bias, which is very common in case-control studies. Another difficulty of case-control studies is the time required to consider exposure factors. Some studies [22, 33] considered exposure factors over 1 year. In the present study, we adopted 6 months preceding the interview to minimize memory bias. Although the exposure time considered in this study was shorter than most previous case-control studies, we interviewed cases immediately after the positive diagnosis of breast cancer to avoid misconceptions about the exposure time. In fact, it represents a great strength of the present study, since in the other studies previously cited [22, 33], cases were interviewed 3-4 months after diagnosis.

We recommend that other breast cancer case-control studies are conducted on populations of higher socioeconomic status from the same region of Brazil. The risk and protective factors published by WCRF and AICR [2] may apply better to such segment of the population, because their lifestyles are more similar to those from developed countries, where most case-control and cohort studies are performed. Furthermore, it is necessary to better explore the question of area of residence to clarify the association of this variable with breast cancer in the present population.

This investigation is the first case-control study on the risk and protective factors for breast cancer in a population from Midwest of Brazil. Findings from our research reinforce the associations between physical activity and family history of breast cancer, in first degree, and breast cancer. Moreover, our results reveal new data that can contribute to elucidate determinant factors for breast cancer, and to prevent this disease in epidemiological transition countries, such as Brazil (Table 6).

## 5. Conclusions

Low per capita income, living in a rural area, and family history of breast cancer are risk factors for breast cancer, whereas practicing physical activity, including at 20 years old, is a protective factor for the disease in women from Midwest of Brazil.

## Figures and Tables

**Table 1:** The inclusion and exclusion criteria of women with breast cancer (cases) and their controls (Midwest, Brazil, 2008–2010).

	Inclusion criteria	Exclusion criteria
Cases	Histopathological breast cancer diagnosis given in the same data or within a week of the interview No prior treatment (e.g., surgery, radiotherapy, chemotherapy) No previous history of cancer in other parts of the body Breast cancer stage III or lower Agreement of signing written informed consent	No breast cancer Already in treatment Previous history of other types of cancer Advanced cancer (stage IV)* Women who had received counseling from a registered dietitian Pregnant or puerperal women Mental or physical disabilities that would hinder the interview

Controls	Age-matched subjects (±5 years) No medical history of cancer Agreement of signing written informed consent	Medical history of breast disease or any type of cancer Women who had received counseling from a registered dietitian Pregnant or puerperal women Mental or physical disabilities that would hinder the interview

*Stage IV cases were excluded because these patients probably would have more pronounced clinical symptoms, such as significant weight change, that could impair data qualify.

**Table 2:** Socioeconomic, gynecological, and anthropometric variables of women with breast cancer (cases) and respective controls, from Midwest of Brazil (2008–2010).

Variable	Cases (*n* = 93)		Controls (*n* = 186)		*P* value*
	Mean	SD	Mean	SD	
Age (years)	51.93	10.07	51.77	9.73	0.897
Height (cm)	1.55	0.05	1.55	0.06	0.570
Current body mass index (BMI) (kg/m²)	27.14	5.44	26.84	4.53	0.623
Waist circumference (cm)	86.94	11.86	86.62	10.15	0.819
Adult weight gain (kg)	16.94	11.65	15.65	10.51	0.416

	Median		Median		*P* value**

Per capita income (US$)	147.00		200.00		0.059
Body mass index (BMI) (kg/m²) at 20 years old	21.91		21.35		0.369
Age at menarche (years)	13		13		0.283
Age at menopause (years)	50		48		0.115
Age at first full pregnancy (years)	21		20		0.099
Number of children	3		3		0.719
Total breastfeeding (months)	24		28		0.557

*Student's *t*-test. **Mann-Whitney test. SD: standard deviation.

**Table 3:** Distribution of socioeconomic and gynecological variables in breast cancer women (cases) and their control subjects, from Midwest of Brazil (2008–2010).

Variable*	Cases (*n* = 93)		Controls (*n* = 186)		Crude OR (95% CI)	*P* value**
	*n*	%	*n*	%		
Age (years)						
<50	40	43.01	72	38.71	1.00	—
≥50	53	56.99	114	61.29	0.84 (0.50–1.39)	0.490
Education level						
Did not study	12	12.90	9	4.84	1.00	—
≤Primary education	59	63.44	133	71.50	0.33 (0.13–0.83)	0.019
≥High school	22	23.66	44	23.66	0.37 (0.14–1.02)	0.056
Per capita income (US$)						
>1/2 Brazilian minimum wage	43	46.24	105	60	1.00	—
≤1/2 Brazilian minimum wage	50	53.76	70	40.00	1.74 (1.05–2.89)	0.032
Area of residence						
Urban	67	83.75	155	96.27	1.00	—
Rural	13	16.25	6	3.73	5.01 (1.82–13.74)	0.002
Bearing children						
Yes	89	95.70	169	90.86	1.00	—
No	4	4.30	17	9.14	0.45 (0.14–1.37)	0.158
Number of children						
Nulliparity	4	4.30	17	9.14	1.00	—
1–3 children	62	66.67	108	58.06	2.44 (0.78–7.58)	0.123
>3 children	27	29.03	61	32.80	1.88 (0.58–6.12)	0.294
Total breastfeeding (months)						
Never	4	4.30	3	1.64	1.00	—
≤6	12	12.90	21	11.48	0.43 (0.08–2.25)	0.316
7–12	10	10.75	20	10.93	0.37 (0.07–2.01)	0.252
>12	67	72.04	139	75.96	0.36 (0.79–1.66)	0.191
Age at first pregnancy (years)						
≤30	83	93.26	155	92.81	1.00	—
>30	6	6.74	12	7.19	0.93 (0.34–2.58)	0.895
Age at menarche (years)						
<12 (early)	13	14.94	28	15.30	1.00	—
≥12 (usual)	74	85.06	155	84.70	1.02 (0.50–2.09)	0.939
Age at menopause (years)						
<55 (usual)	34	91.89	111	92.50	1.00	—
≥55 (late)	3	8.11	9	7.50	1.09 (.28–4.25)	0.903
Family history of cancer						
No	30	32.26	94	50.54	1.00	—
Yes	63	67.74	92	49.46	2.14 (1.27–3.61)	0.004
Family history of breast cancer (mother/sisters)						
No	85	91.40	182	97.85	1.00	—
Yes	8	8.60	4	2.15	4.28 (1.25–14.61)	0.020

OR: odds ratio and CI: confidence interval.

*Per capita income: Brazilian minimum wage corresponds to approximately US$ 300; 11 control subjects did not indicate their per capita income; area of residence: information is unavailable for 13 cases and 25 controls; total breastfeeding: 3 controls did not indicate their breastfeeding time; age at first pregnancy: 4 cases and 19 controls did not have children or did not indicate their age at first pregnancy; age at menarche: 6 cases and 3 controls did not indicate their age at menarche; age at menopause: 12 cases and 11 controls did not indicate their age at menopause; 44 cases and 55 controls were premenopausal.

**Wald statistic.

**Table 4:** Distribution of anthropometric and lifestyle variables in breast cancer women (cases) and control subjects, from Midwest of Brazil (2008–2010).

Variable*	Cases (*n* = 93)		Controls (*n* = 186)		Crude OR (95% CI)	*P* value**
	*n*	%	*n*	%		
Adult weight gain (kg)						
≤5	10	14.08	14	9.52	1.00	—
5.01–10	11	15.49	33	22.45	0.47 (0.16–1.35)	0.159
>10	50	70.43	100	68.03	0.71 (0.29–1.71)	0.441
Current nutritional status						
Eutrophic	38	40.86	70	37.63	1.00	—
Preobesity	29	31.18	71	38.17	0.75 (0.42–1.35)	0.341
Obesity	26	27.96	45	24.19	1.06 (0.57–1.98)	0.845
Nutritional status (at 20 years old)						
Eutrophic	67	94.37	134	91.16	1.00	—
Overweight	4	5.63	13	8.84	0.61 (0.19–1.96)	0.411
Waist circumference (cm)						
<80	29	31.18	47	25.27	1.00	—
≥80	64	68.82	139	74.73	0.75 (0.43–1.29)	0.296
Height (cm)						
<160	78	83.87	138	74.19	1.00	—
≥160	15	16.13	48	25.81	0.55 (0.29–1.05)	0.071
Alcohol beverage consumption						
Absent or moderate	90	97.83	184	98.92	1.00	—
Excessive	2	2.17	2	1.08	2.04 (0.28–14.75)	0.478
Smoking habit						
Not smoker	53	56.99	108	58.06	1.00	—
Ex-smoker	26	27.96	46	24.73	1.15 (0.64–2.06)	0.634
Smoker	14	15.05	32	17.20	0.89 (0.44–1.81)	0.751
Physical activity (past 6 months)						
Sedentary	34	36.56	24	12.90	1.00	—
Irregularly active B	16	17.20	33	17.74	0.34 (0.15–0.76)	0.008
Irregularly active A	9	9.68	39	20.97	0.16 (0.07–0.40)	0.000
Active	34	36.56	90	48.39	0.27 (0.14–0.51)	0.000
Occupational physical activity (at 20 years old)						
No	57	72.15	99	61.88	1.00	—
Yes	22	27.85	61	38.13	0.62 (0.35–1.12)	0.118
Housework physical activity (at 20 years old)^f^						
No	13	16.46	20	12.50	1.00	—
Yes	66	83.54	140	87.50	0.72 (0.34–1.55)	0.406
Leisure physical activity (at 20 years old)^f^						
No	74	93.67	119	74.38	1.00	—
Yes	5	6.33	41	25.62	0.20 (0.07–0.52)	0.001

OR: odds ratio and CI: confidence interval.

*Adult weight gain: 22 cases and 39 controls did not indicate their weight at 20 years old; current nutritional status: eutrophic—body mass index (BMI) = 18.4 kg/m² to 24.9 kg/m²; preobesity—BMI = 25 kg/m² to 29.9 kg/m²; obesity—BMI ≥ 30 kg/m² [11]; alcohol beverage consumption: 1 case did not indicate alcohol beverage intake; occupational physical activity: information unavailable for 14 cases and 26 controls.

**Wald statistic.

**Table 5:** Per capita income, area of residence, physical activity, and family history of breast cancer of women with breast cancer (cases) and respective controls, from Midwest of Brazil (2008–2010).

Variable*	Cases (*n* = 93)		Controls (*n* = 186)		Adjusted OR (95% CI)	*P* value**
	*n*	%	*n*	%		
*First level*						
Per capita income (US$)						
>1/2 Brazilian minimum wage	43	46.24	105	60.00	1.00	—
≤1/2 Brazilian minimum wage	50	53.76	70	40.00	1.87 (1.06–3.29)	0.031
Area of residence						
Urban	67	83.75	155	96.27	1.00	—
Rural	13	16.25	6	3.73	4.93 (1.65–14.73)	0.004
*Second level*						
Family history of breast cancer						
No	85	91.40	182	97.85	1.00	—
Yes	8	8.60	4	2.15	5.38 (1.46–19.93)	0.012
Physical activity (past 6 months)						
Sedentary	34	36.56	24	12.90	1.00	—
Irregularly active B	16	17.20	33	17.74	0.50 (0.18–1.37)	0.178
Irregularly active A	9	9.68	39	20.97	0.26 (0.09–0.77)	0.016
Active	34	36.56	90	48.39	0.23 (0.10–0.55)	0.001
Leisure physical activity (at 20 years old)						
No	74	93.67	119	74.38	1.00	—
Yes	5	6.33	41	25.62	0.13 (0.03–0.54)	0.005

OR: odds ratio and CI: confidence interval; multivariate analyses adjusted for educational level, per capita income, area of residence, bearing children, number of children, height, physical activity (past 6 months), previous occupational and leisure physical activity, family history of cancer (any type), and family history of breast cancer (mother/sisters).

*Per capita income: Brazilian minimum wage corresponds to approximately US$ 300, 11 control subjects did not indicate their per capita income; area of residence: information is unavailable for 13 cases and 25 controls.

**Wald statistic.

**Table 6:** Findings from the case-control study (2011) of Midwest, Brazil, compared to World Cancer Research Fund and American Institute for Cancer Research evidences (2007) [2].

New data	Reinforce the literature	Not confirmed
Low per capita income (risk factor)	Physical activity (protective factor)	Breastfeeding
Residence in rural area (risk factor)	Family history of breast cancer in first degree (risk factor)	Gynecological variables (bearing children, age at first pregnancy, age at menarche, age at menopause)
		Anthropometric variables (adult weight gain, nutritional status, waist circumference, height)
		Alcohol beverage consumption
		Smoking habit

## References

[1] Ministério da Saúde, Instituto Nacional de Câncer (2009). *Políticas e Ações Para Prevenção do Câncer no Brasil: Alimentação, Nutrição e Atividade Física*.

[2] World Cancer Research Fund (WCRF), American Institute for Cancer Research (AICR) (2007). *Food, Nutrition, Physical Activity, and the Prevention of Cancer: A Global Perspective*.

[3] Kelsey JL (1993). Breast cancer epidemiology: summary and future directions. *Epidemiologic Reviews*.

[4] Ministério da Saúde (Brasil) (2009). *Estimativa 2010: Incidência de Câncer no Brasil*.

[5] Schmidt ME, Steindorf K, Mutschelknauss E (2008). Physical activity and postmenopausal breast cancer: effect modification by breast cancer subtypes and effective periods in life. *Cancer Epidemiology Biomarkers and Prevention*.

[6] Peplonska B, Lissowska J, Hartman TJ (2008). Adulthood lifetime physical activity and breast cancer. *Epidemiology*.

[7] Bissonauth V, Shatenstein B, Fafard E (2009). Weight history, smoking, physical activity and breast cancer risk among French-Canadian women non-carriers of more frequent BRCA1/2 mutations. *Journal of Cancer Epidemiology*.

[8] Slattery ML, Edwards S, Murtaugh MA (2007). Physical activity and breast cancer risk among women in the Southwestern United States. *Annals of Epidemiology*.

[9] Frenk J (1991). The epidemiological transition in Latin America. *Boletin de la Oficina Sanitaria Panamericana*.

[10] Hanf V, Gonder U (2005). Nutrition and primary prevention of breast cancer: foods, nutrients and breast cancer risk. *European Journal of Obstetrics Gynecology and Reproductive Biology*.

[11] World Health Organization (2000). Obesity: preventing and managing the global epidemic. *WHO Technical Report Series*.

[12] Brandt B, Hermann S, Straif K, Tidow N, Buerger H, Chang-Claude J (2004). Modification of breast cancer risk in young women by a polymorphic sequence in the egfr gene. *Cancer Research*.

[13] Peixoto MDRG, Monego ET, Alexandre VP, De Souza RGM, De Moura EC (2008). Surveillance of risk factors for chronic diseases through telephone interviews: experience in Goiânia, Goiás State, Brazil. *Cadernos de Saúde Pública*.

[14] Lahmann PH, Schulz M, Hoffmann K (2005). Long-term weight change and breast cancer risk: the European prospective investigation into cancer and nutrition (EPIC). *British Journal of Cancer*.

[15] Lohman TG, Roche AF, Martorell R (1988). *Anthropometric Standardization Reference Manual*.

[16] Habicht JP (1974). Estandarizacion de metodos epidemiologicos cuantitativos sobre el terreno. *Boletín de la Oficina Sanitaria Panamericana*.

[17] Brown LM, Gridley G, Wu AH (2010). Low level alcohol intake, cigarette smoking and risk of breast cancer in Asian-American women. *Breast Cancer Research and Treatment*.

[18] Loguercio C, Tuccillo C, Federico A, Fogliano V, Del Vecchio Blanco C, Romano M (2009). Alcoholic beverages and gastric epithelial cell viability: effect on oxidative stress-induced damage. *Journal of Physiology and Pharmacology*.

[19] Matsudo SM, Matsudo VR, Araújo T (2002). Physical activity level of São Paulo State population: an analysis based on gender, age, socio-economic status, demographics and knowledge. *Revista Brasileira de Ciência e Movimento*.

[20] Magnusson C, Wedrén S, Rosenberg LU (2007). Cigarette smoking and breast cancer risk: a population-based study in Sweden. *British Journal of Cancer*.

[21] Shahar S, Salleh RM, Ghazali AR, Koon PB, Wan Mohamud WN (2010). Roles of adiposity, lifetime physical activity and serum adiponectin in occurrence of breast cancer among Malaysian women in Klang Valley. *Asian Pacific Journal of Cancer Prevention*.

[22] Bessaoud F, Daurès JP (2008). Patterns of alcohol (especially wine) consumption and breast cancer risk: a case-control study among a population in Southern France. *Annals of Epidemiology*.

[23] Sanderson M, Peltz G, Perez A (2010). Diabetes, physical activity and breast cancer among Hispanic women. *Cancer Epidemiology*.

[24] Steel RG, Torrie JH (1996). *Principles and Procedures of Statistics: A Biometrical Approach*.

[25] Instituto Btasileiro de Geografia e Estatística (IBGE) Síntese de indicadores sociais (2008). http://www.ibge.gov.br/home/estatistica/populacao/condicaodevida/indicadoresminimos/sinteseindicsociais2008/default.shtm.

[26] Instituto Brasileiro de Geografia e Estatística (IBGE) Pesquisa de orçamentos familiares 2008-2009. http://www.ibge.gov.br/home/xml/pof_2008_2009.shtm.

[27] Peres F, Rozemberg B, de Lucca SR (2005). Risk perception related to work in a rural community of Rio de Janeiro State, Brazil: pesticides, health, and environment. *Cadernos de Saúde Pública*.

[28] Palacín L, Moraes MAS (1994). *História de Goiás*.

[29] Huicho L, Trelles M, Gonzales F, Mendoza W, Miranda J (2009). Mortality profiles in a country facing epidemiological transition: an analysis of registered data. *BMC Public Health*.

[30] Tessaro S, Béria JU, Tomasi E, Victora CG (2003). Breastfeeding and breast cancer: a case-control study in Southern Brazil. *Cadernos de Saúde Pública*.

[31] Knudson AG (2001). Two genetic hits (more or less) to cancer. *Nature Reviews Cancer*.

[32] World Health Organization (WHO) (2004). *Global Strategy on Diet, Physical Activity and Health*.

[33] Berstad P, Ma H, Bernstein L, Ursin G (2008). Alcohol intake and breast cancer risk among young women. *Breast Cancer Research and Treatment*.

